# Superior labrum anterior to posterior lesions: Part 2 – Classification with arthroscopic correlation

**DOI:** 10.4102/sajr.v27i1.2707

**Published:** 2023-11-28

**Authors:** Peter Mercouris, Matthew Mercouris

**Affiliations:** 1 Diagnotic Radiologist, Lake, Smit and Partners, Gateway Private Hospital, Durban, South Africa; 2 Department of Orthopaedic, Mitchell’s Plain District Hospital, Cape Town, South Africa

**Keywords:** shoulder, glenoid labrum, MRI, arthroscopy, anatomic variants, SLAP lesion or tear

## Abstract

The glenoid labrum deepens the glenoid fossa and allows for the attachment of the long head of the biceps tendon and glenohumeral ligaments, contributing to the stability of the glenohumeral joint. The superior labrum is a common site of labral injury. The acronym SLAP (superior labrum anterior to posterior or anteroposterior) lesion was introduced by Snyder and colleagues in 1990 to describe superior labral tears based on arthroscopic evaluation. This original classification has since been expanded, and there are currently 10 types of SLAP lesions. The article will describe and illustrate the 10 types of SLAP lesions by means of colour illustrations, MRI images and correlative arthroscopy images. A practical approach to the assessment of SLAP lesions will be recommended.

**Contribution:**

The illustrated review functions as a crucial radiological guide for both radiologists and orthopaedic surgeons. The combination of illustrations, MR and correlative arthroscopic images enhances the comprehensive understanding of labral pathology. The value of the review lies in the presentation of imaging findings and classification, coupled with findings on arthroscopy. This understanding is vital in guiding orthopaedic management for patients, ensuring appropriate treatment strategies.

## Introduction

In 1985, Andrews and colleagues described superior labral injuries in throwing athletes.^1^ This was followed by Snyder and colleagues in 1990, who introduced the term or acronym SLAP (superior labrum anterior to posterior or anteroposterior) to describe injuries of the superior labrum and, based on arthroscopic evaluation, classified SLAP lesions of the shoulder into four types.^2^ Following the identification of additional SLAP tears, the original classification has been revised, and there are presently 10 types of SLAP lesions.^3,4^ This review pictorially illustrates the spectrum of SLAP lesions by means of colour illustrations, MRI images and correlative arthroscopy images. The clinical presentation, diagnostic and management challenges are highlighted.

## Diagnostic evaluation

The clinical diagnosis of a SLAP lesion is difficult, and patients often present with non-specific shoulder pain particularly with overhead or cross-body motion. The patient may also complain of popping, clicking, catching weakness, stiffness and instability. The presentation may be acute with the mechanism of injury involving a traction injury, direct trauma to the shoulder or commonly a fall on an outstretched arm, or it may be chronic because of repetitive microtrauma secondary to overhead activity.^5,6^ There is no single physical test or sign that is specific for a SLAP lesion,^5^ and clinical assessment is further complicated by the strong association with co-existent shoulder injuries such as rotator cuff tears, Bankart lesions and glenohumeral articular damage.^7^

Arthroscopy remains the gold standard for diagnosis of a SLAP lesion, but MRI, particularly direct MR arthrography that involves the intra-articular administration of gadolinium-based contrast medium, has been shown to be accurate in the evaluation of the glenoid labrum.^5,8^

## Classification

There are 10 different types of SLAP lesions, as outlined in Table 1.^2,3,9,10^ The arthroscopic prevalence of SLAP lesions in previous studies ranged from 3.9% to 11.8%^2,9^, but a more recent study has shown the prevalence to be as high as 26%.^11^ Snyder’s original classification of SLAP lesions described the first four main types.^2^

**TABLE 1:** Classification of SLAP lesions.

Type	Description
I	Fraying of superior labrum
II	Tear of superior labrum with biceps tendon extension. Subdivided into: Type A: Tear extending into anterosuperior labrum Type B: Tear extending into posterosuperior labrum Type C: Tear extending into anterosuperior and posterosuperior labrum
III	Bucket handle tear of superior labrum with intact biceps tendon
IV	Bucket handle tear of superior labrum with biceps tendon extension
V	Superior labral tear with anteroinferior extension or Bankart lesion with superior extension
VI	Flap tear of superior labrum
VII	Superior labral tear with extension into middle glenohumeral ligament
VIII	Superior labral tear with extension into posterior labrum (more extensive than type IIB)
IX	Circumferential labral tear
X	Superior labral tear with extension into rotator interval through superior glenohumeral ligament

*Source*: Please see the full reference list of the article Snyder SJ, Karzel RP, Del Pizzo W, et al. SLAP lesions of the shoulder. Arthroscopy. 1990;6(4):274–279. https://doi.org/10.1016/0749-8063(90)90056-J, for more information

In 1991, Morgan and colleagues subdivided the type II lesion into subtypes A, B and C with the type A lesion being primarily anterior, the type B lesion primarily posterior and the type C lesion a combination of anterior and posterior tears.^11^ Maffett and colleagues subsequently identified a further three types of SLAP lesions, types V, VI and VII.^9^

Between 1997 and 2000, an additional three categories of SLAP lesions (VIII, IX and X) were introduced in informal talks, small meetings and conferences.^3^ There is another type of SLAP X lesion that exists in the literature, and this represents a superior labral tear with an associated reverse Bankart lesion.^12^

## Imaging approach

The labrum, biceps tendon and glenohumeral ligaments should be considered as a single functional unit,^8^ highlighting the importance of a comprehensive evaluation of the labrum and adjacent structures. This concept arose primarily from the macroscopic and microscopic study by Huber and Putz^13^ that identified the periarticular fiber system, comprising bundles of parallel collagen fibers that run around the entire circumference of the glenoid and into the adjacent structures including the biceps tendon and glenohumeral ligaments. There is, however, individual variability of the periarticular fiber system because of the varied origin of the long head of the biceps tendon and the morphology of the glenohumeral ligaments. This patient variability most likely predisposes to certain patterns of injury, given an identical mechanism.^8^

MR arthrography is a highly effective method for the detection of SLAP lesions but less so in the classification of the different types of SLAP lesions with 66% concurrence between MR arthrography and arthroscopy grading.^14^ A practical approach to the assessment of the labrum for a suspected SLAP lesion is therefore recommended, which also reinforces the concept of the periarticular fiber system and single functional unit and, should entail the evaluation of the following: (1) biceps labral complex for a tear, (2) the morphology of the tear (non-displaced or displaced), (3) the extension pattern of the tear to involve other parts of the labrum, (4) the glenohumeral ligaments and (5) the associated involvement of the regional structures that include the joint capsule, osteochondral elements and rotator cuff tendons.

The labrum should be assessed for its size, shape, signal intensity and attachment to the glenoid. The size is variable and of no practical diagnostic value.^15,16^ The shape is also variable and is most commonly triangular^17^ but can also be rounded, blunted, cleaved, notched, crescent-shaped and even absent.^17,18^ The labrum usually has regular margins and is of low signal on all MR pulse sequences. The labral attachment to the glenoid is extremely variable, particularly in the superior and anterosuperior quadrants, and this poses the greatest diagnostic challenge in differentiating developmental detachment from pathological detachment. There are five anatomic variants in this region, namely the sublabral recess or sulcus, sublabral foramen or hole, Buford complex, bicipital labral sulcus or pseudo-SLAP tear and the high attachment of the anterior band of the glenohumeral ligament. These may all be mistaken for labral tears, especially the sublabral recess.

The diagnosis of SLAP lesions on MRI is based on the two primary signs of abnormal labral morphology and linear high signal intensity (fluid or contrast) in the labrum. A paralabral cyst is a secondary sign of a labral tear and is typically located in the spinoglenoid notch. These cysts may not fill during MR arthrography (MRA), as direct communication between the cyst and glenohumeral joint rarely occurs, emphasising the importance of obtaining a T2-weighted sequence as part of the routine MRA protocol (Figure 13d and Figure 13e).

It is important to assess whether the superior labral tear extends into the biceps tendon and to characterise the extent of biceps involvement as a compromise of the biceps anchor may determine the need for surgical treatment with biceps anchor stabilisation. The morphology of the labral tear should be evaluated as the labrum may remain in its normal location (non-displaced) or be displaced, having a fragment that has lost connection with the parent labrum and includes a bucket handle or flap tear.

## Imaging diagnosis

**FIGURE 1:**
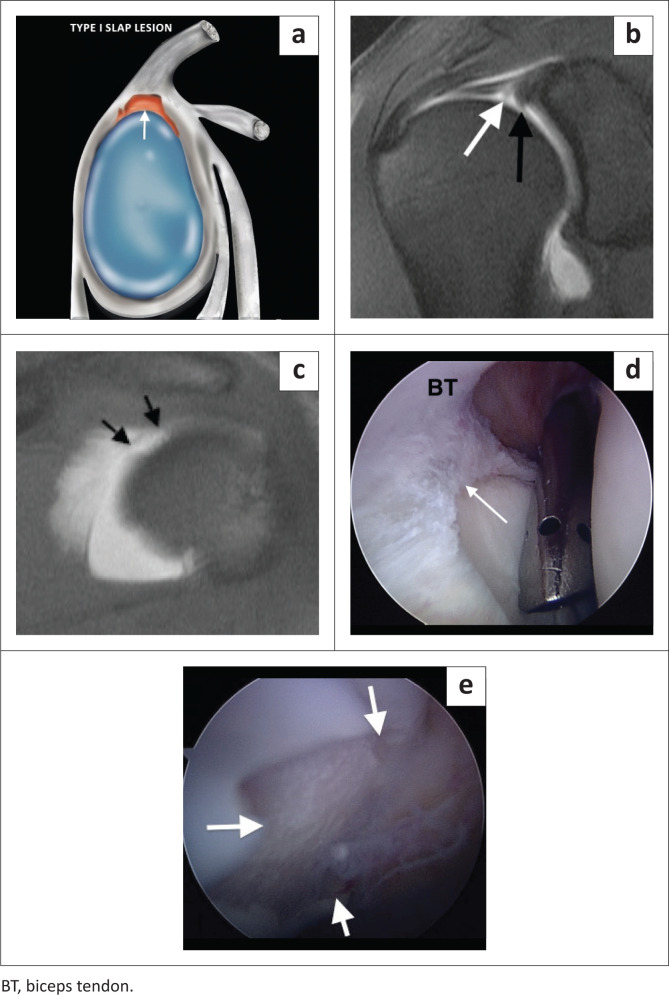
A SLAP type I lesion. (a) Colour illustration. (b) Degenerative fraying (white arrow) of the superior labrum. Note the high attachment of the inferior glenohumeral ligament (black arrow). (c) FS T1-weighted MR arthrogram coronal image demonstrating an articular cartilage defect (between the black arrows) in the superomedial portion of the humeral head. (d) Arthroscopic confirmation of fraying of the superior labrum (white arrow). (e) Arthroscopic confirmation of a chondral fracture (white arrows).

The type I SLAP lesion represents fraying of the superior labrum with an intact biceps anchor. This lesion is common in athletes with repetitive overhead shoulder activity but can also be seen incidentally as part of normal age-related labral degeneration.^3^ The fraying of the superior labrum may be difficult to appreciate, and the morphologic abnormality is probably best shown on direct MR arthrography (Figure 1).^4^

**FIGURE 2:**
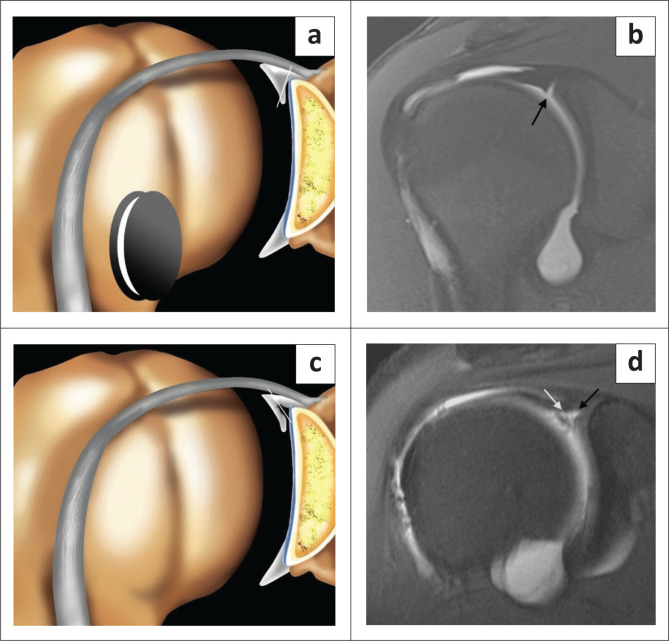
Sublabral recess versus SLAP tear. (a) Colour illustration of a sublabral recess or sulcus – white line (or recess) is medially oriented. (b) FS T1 coronal oblique arthrogram demonstrating medial orientation of contrast-filled recess. (c) Colour illustration of SLAP tear – white line of tear is laterally oriented. (d) FS T1 coronal oblique arthrogram demonstrating the lateral orientation of the contrast-filled tear.

**FIGURE 3:**
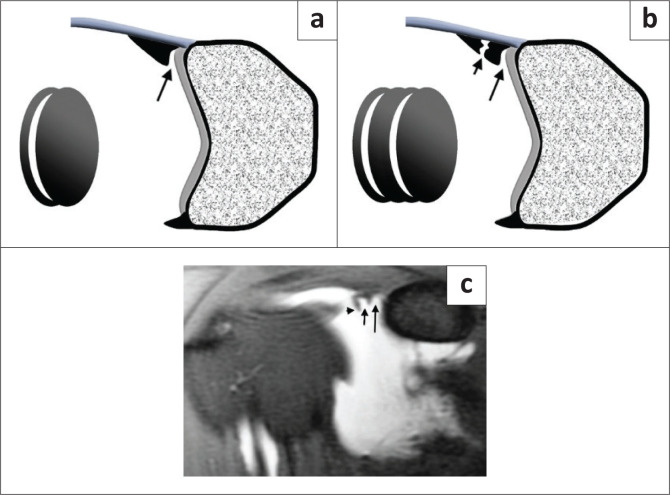
‘Oreo cookie’ sign. (a) Illustration showing a single ‘Oreo cookie’ in a sublabral recess (black arrow). (b) Illustration showing a double ‘Oreo cookie’ sign in a SLAP III tear. Long black arrow – sublabral recess, short black arrow – contrast (or fluid) filled defect of the displaced labral tear. (c) FS T1-weighted MR arthrogram coronal oblique image reflecting the illustration in (b) in this patient with an arthroscopically proven type III SLAP tear. The long black arrow represents the sublabral recess and the short black arrow the contrast-filled defect of the displaced labral tear (black arrowhead).

**FIGURE 4:**
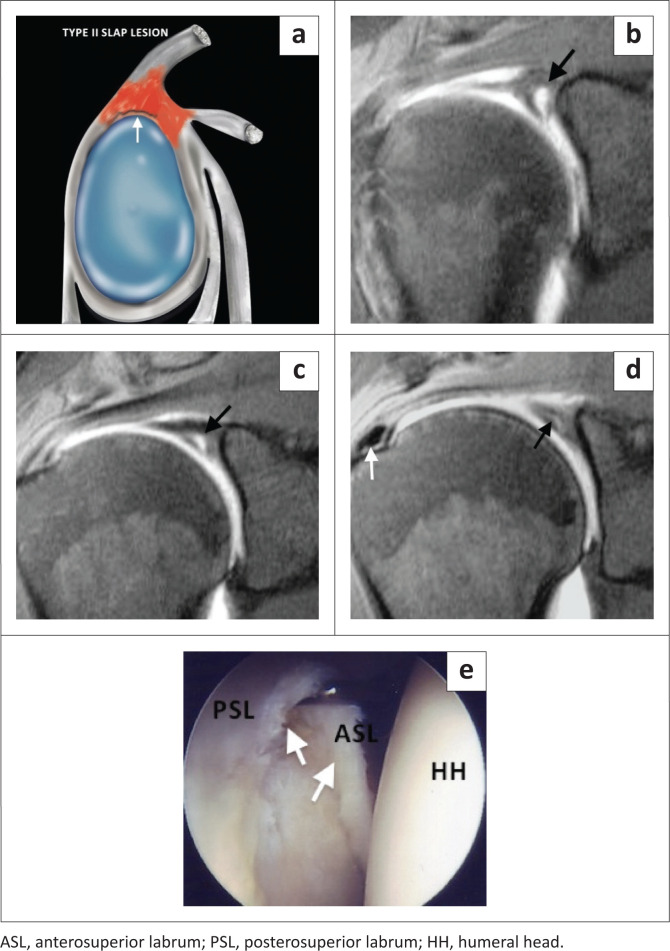
A SLAP type II lesion. (a) Colour illustration. (b) FS T1-weighted (T1W) MR arthrogram coronal oblique image demonstrating an enlarged (> 5 mm) globular appearing sublabral contrast collection indicative of a superior labral tear (black arrow). (c) FS T1W MR arthrogram coronal oblique image depicting a laterally oriented contrast collection (black arrow), a feature of a labral tear. (d) FS T1W MR arthrogram coronal oblique image posterior to image (c) confirms the extension of the superior labral tear posterior to the biceps anchor. White arrow shows incidental calcification in the infraspinatus tendon. (e) Arthroscopic (posterior portal) confirmation of a type II lesion. The probe elevates the torn and detached superior labrum (white arrows).

**FIGURE 5:**
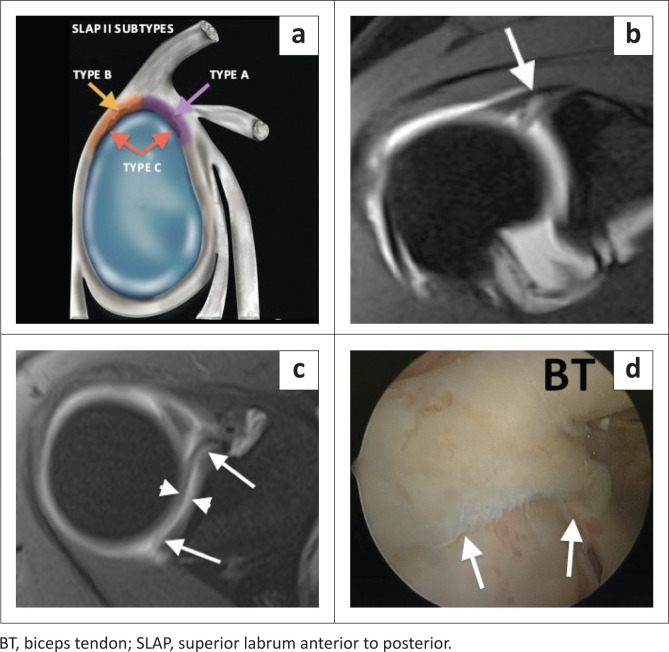
The SLAP II lesion subtypes. (a) Colour illustration demonstrating the SLAP II subtypes: (1) Type A – anterior SLAP II lesion (purple shading and arrow). (2) Type B – posterior SLAP II lesion (orange shading and arrow). (3) Type C – classic type II SLAP lesion with anterior and posterior extension. (b) FS T1-weighted (T1W) MR arthrogram coronal oblique image demonstrating a superior labral tear with biceps anchor involvement (white arrow). (c) FS T1W MR arthrogram axial image depicting the full anterior to posterior extent (white arrows) of the superior labral tear and detachment (white arrowheads) indicating a type C SLAP II tear. (d) Arthroscopic (posterior portal) confirmation of the SLAP II tear (white arrows).

The type II SLAP lesion is a tear of the superior labrum with biceps anchor extension. The type II lesion is also common in athletic overhead activity and represents the most common type of SLAP lesion at arthroscopy and on MRI with a reported incidence of 41% – 55%.^19^ The sublabral recess variant may be mistaken for a type II SLAP lesion, and both entities can have the appearance of a single ‘Oreo cookie’ on coronal oblique images^3^ (Figure 2a and Figure 3a). Signs that are helpful in supporting the diagnosis of a tear rather than a normal recess include: a laterally oriented high signal intensity in the coronal oblique plane (Figure 2c and Figure 4c), extension of the high signal posterior to the biceps anchor in the coronal oblique plane (Figure 4d), a recess greater than 2 mm in size in its mediolateral dimension as measured in the coronal oblique plane (Figure 4b), anteroposterior extension of the high signal intensity in the axial plane (Figure 5c) and paralabral cyst formation.^3,4,19,20^ The anterior SLAP II lesion is associated with an anterior supraspinatus articular-sided partial thickness tendon tear in the SLAC (superior labrum anterior cuff) lesion and the posterior SLAP II lesion is the posterior peel-back lesion seen in the throwing athlete (Figure 5a).^4^

**FIGURE 6:**
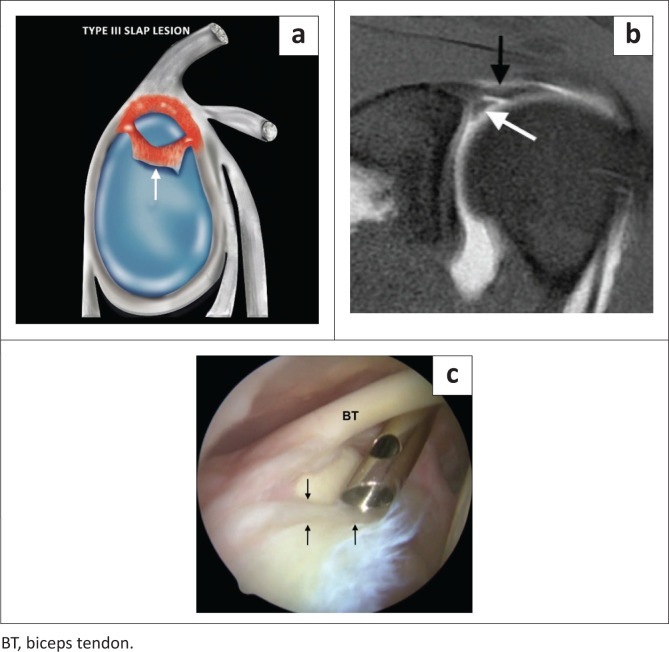
A SLAP type III lesion. (a) Lateral colour illustration of a SLAP III lesion. (b) FS T1-weighted (T1W) MR arthrogram coronal oblique image demonstrating an inferiorly displaced superior labrum (white arrow) consistent with a bucket handle tear but with an intact biceps anchor (black arrow). (c) Arthroscopic probe displacing the bucket handle component inferiorly (black arrows).

**FIGURE 7:**
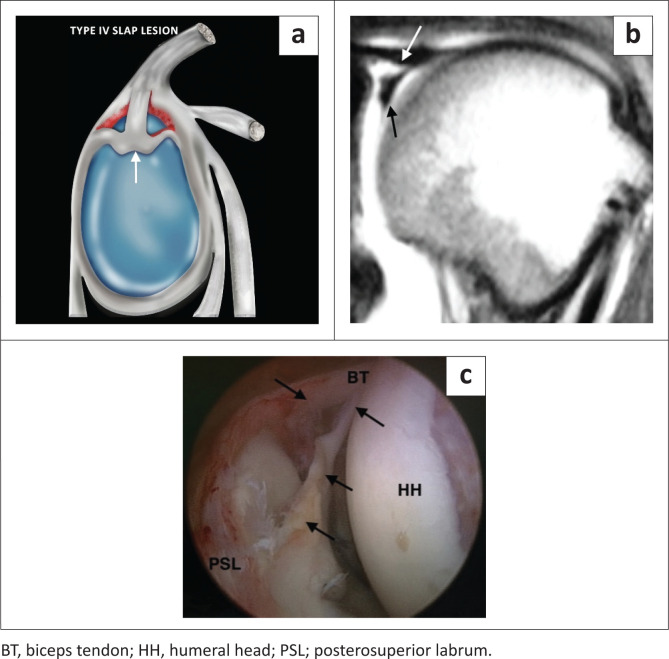
A SLAP type IV lesion. (a) Lateral colour illustration of a SLAP IV tear. (b) FS T1-weighted (T1W) MR arthrogram coronal image showing inferior displacement of the torn superior labrum (black arrow) and involvement of the biceps anchor (white arrow). (c) Arthroscopic image confirming the bucket handle tear extending into the biceps anchor (black arrows).

Type III SLAP lesion is a bucket handle tear of the superior labrum with an intact biceps anchor (Figure 6), whereas the type IV SLAP lesion represents a bucket handle tear of the superior labrum with biceps anchor extension (Figure 7), and both are usually the result of a fall on an outstretched arm.

**FIGURE 8:**
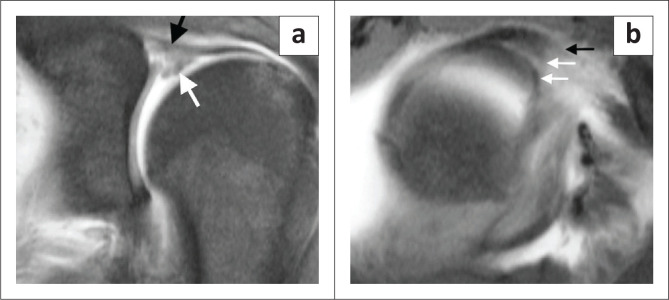
‘Triple structure’ sign. (a) FS T1-weighted (T1W) MR arthrogram coronal image showing inferior displacement of the torn superior labrum (white arrow) and involvement of the biceps anchor (black arrow) of an arthroscopically confirmed SLAP type IV tear. (b) FS T1W MR arthrogram sagittal oblique image demonstrating the ‘three structure’ sign. Black arrow depicts the biceps tendon and white arrows depict the bucket handle fragments.

The type III and IV lesions are characterised by a bucket handle tear, and this is seen as displaced labral tissue on the coronal oblique and sagittal oblique images. Two useful diagnostic signs have been described: the double ‘Oreo cookie’ sign and the ‘triple structure’ sign. The double ‘Oreo cookie’ sign refers to the presence of a sublabral recess and a displaced labral fragment on a coronal oblique image (Figure 3).^3^ The ‘triple structure’ or ‘three structure’ sign refers to three separate and distinct structures that may be seen on sagittal or coronal oblique images. Contrast (or fluid) is seen between the biceps tendon and the superior labrum as well as between the two separated labral fragments (Figure 8).^4^

**FIGURE 9:**
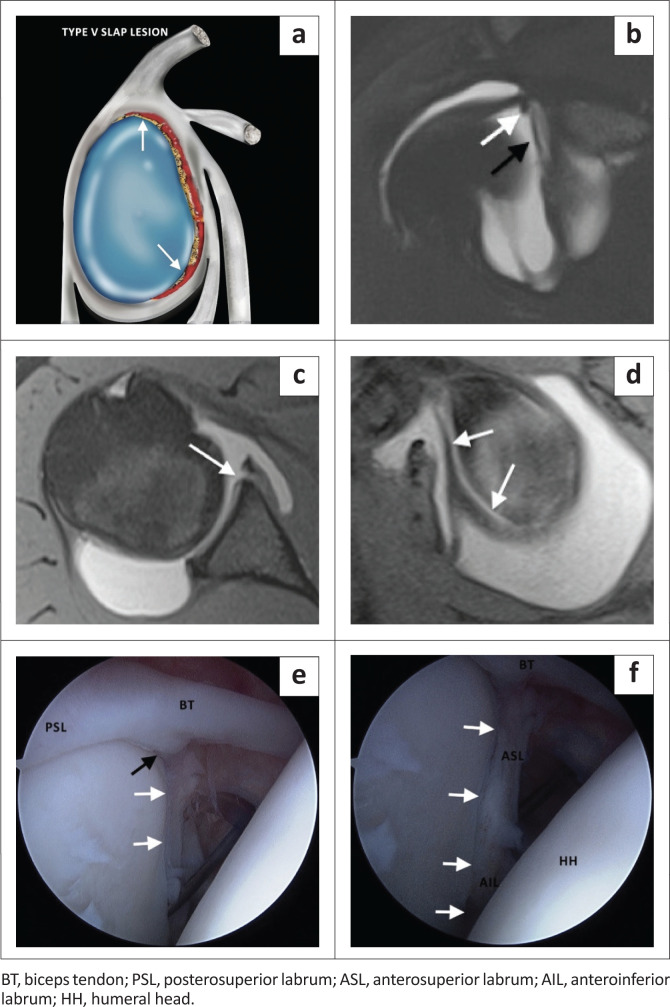
A SLAP type V lesion. (a) Lateral colour illustration of a SLAP V tear. (b) FS T2-weighted (T2W) coronal oblique (post arthrogram) image demonstrating abnormal superior labral morphology (white arrow) and anterosuperior labral detachment (black arrow). (c) FS T1-weighted (T1W) post arthrogram axial image showing the anteroinferior labral tear (white arrow) of a Bankart lesion. (d) FS T1W post arthrogram sagittal image depicting detachment of entire anterior labrum (white arrows). (e) Arthroscopic (posterior portal) image confirming the superior labral tear (black arrow) and detachment of anterosuperior labrum (white arrows). (f) Arthroscopic image showing the tear extending to involve the entire anterior labrum (white arrows).

The type V SLAP lesion is a superior labral tear with anteroinferior extension or a Bankart lesion with superior extension and is usually associated with an anterior dislocation of the shoulder. The SLAP component of the tear is best visualised in the coronal oblique plane (Figure 9b), whereas the Bankart component is best seen in the axial plane (Figure 9c) and in the abduction and external rotation (ABER) position. A Hill-Sachs lesion may also be seen in the axial plane above the level of the coracoid process.

**FIGURE 10:**
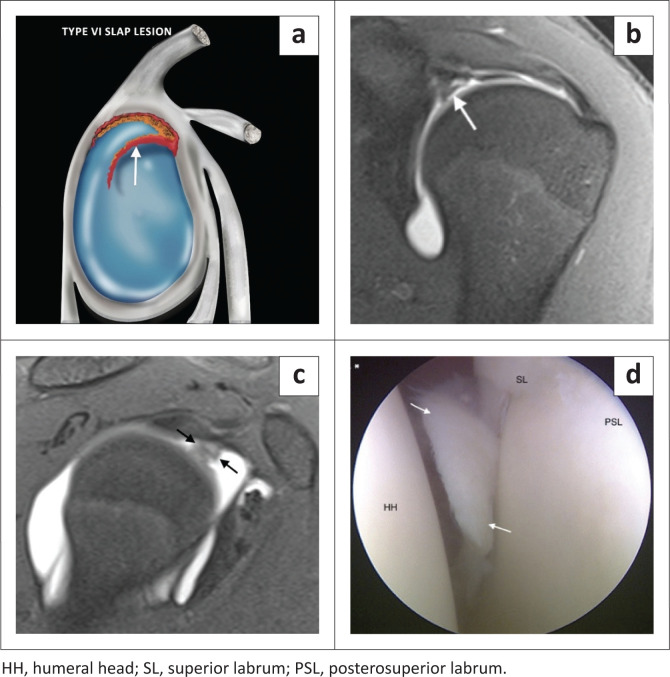
A SLAP type VI lesion. (a) Lateral colour illustration of a SLAP VI tear. (b) FS T1-weighted (T1W) MR arthrogram coronal image displaying the displaced labrum (white arrow). (c) FS T1 MR arthrogram sagittal image displaying the anteriorly displaced flap of labral tissue (black arrows). (d) Arthroscopic (posterior portal) image confirming the anterior flap (white arrows).

The type VI SLAP lesion represents a flap tear of the superior labrum and probably represents a tear of the bucket handle component of a type III or IV lesion.^3^ The flap tear can either be anterior (Figure 10) or posterior and is usually seen after a fall on an outstretched arm. This can be a very difficult diagnosis to make and is best identified on coronal images (Figure 10b) although sagittal images (Figure 10c) are required to appreciate the flap component.^4^

**FIGURE 11:**
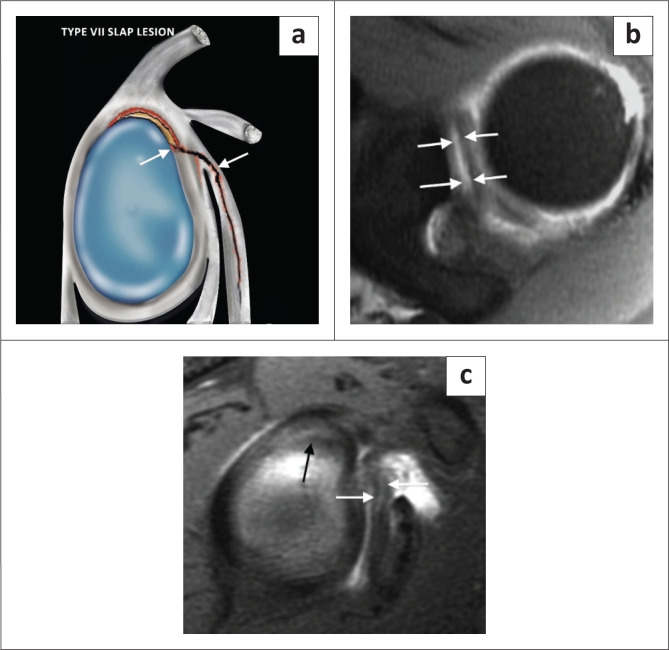
A SLAP type VII lesion. (a) Lateral colour illustration of a SLAP VII tear. (b) FS T1-weighted (T1W) MR arthrogram axial image showing a superior labral tear extending from anterior to posterior (white arrows). (c) FS T1W MR arthrogram sagittal image demonstrating extension of this superior labral tear (black arrow) into the middle glenohumeral ligament (white arrows) in this arthroscopically confirmed SLAP VII tear.

The type VII SLAP lesion is a superior labral tear with extension into the middle glenohumeral ligament (Figure 11) and, as with SLAP V lesions, is commonly associated with anterior shoulder dislocation.

**FIGURE 12:**
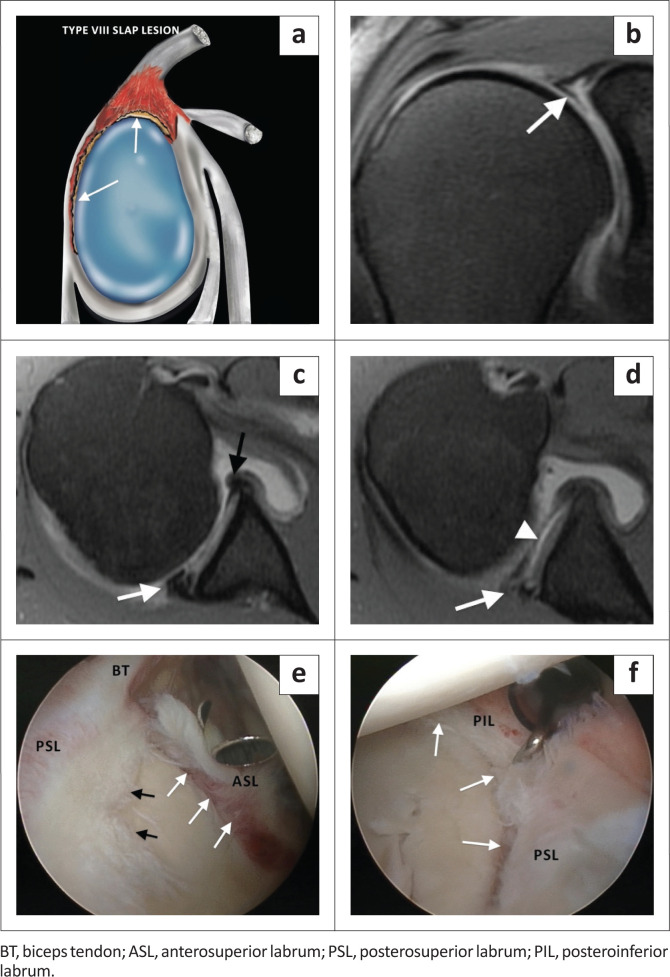
A SLAP type VIII lesion. (a) Lateral colour illustration of a SLAP VIII tear. (b) FS T1-weighted (T1W) MR arthrogram coronal oblique image showing the complex tear pattern of the superior labrum including the biceps anchor (white arrow). (c) FS T1W MR arthrogram axial image below the level of the coracoid process depicting the posteroinferior extension of the labral tear. White arrow – torn posterior labrum and black arrow – normal anterior labrum. (d) FS T1W MR arthrogram axial image at the level of the inferior joint indicating that the tear extends to involve the inferior labrum at the 6 o’clock position. White arrow denotes the torn posteroinferior labrum and white arrowhead the detachment of the inferior labrum. (e) Arthroscopy confirming the superior labral tear with anterior (white arrows) and posterior (black arrows) extension. (f) Arthroscopic view with white arrows depicting the torn and detached posterosuperior (PSL) and posteroinferior labrum (PIL).

**FIGURE 13:**
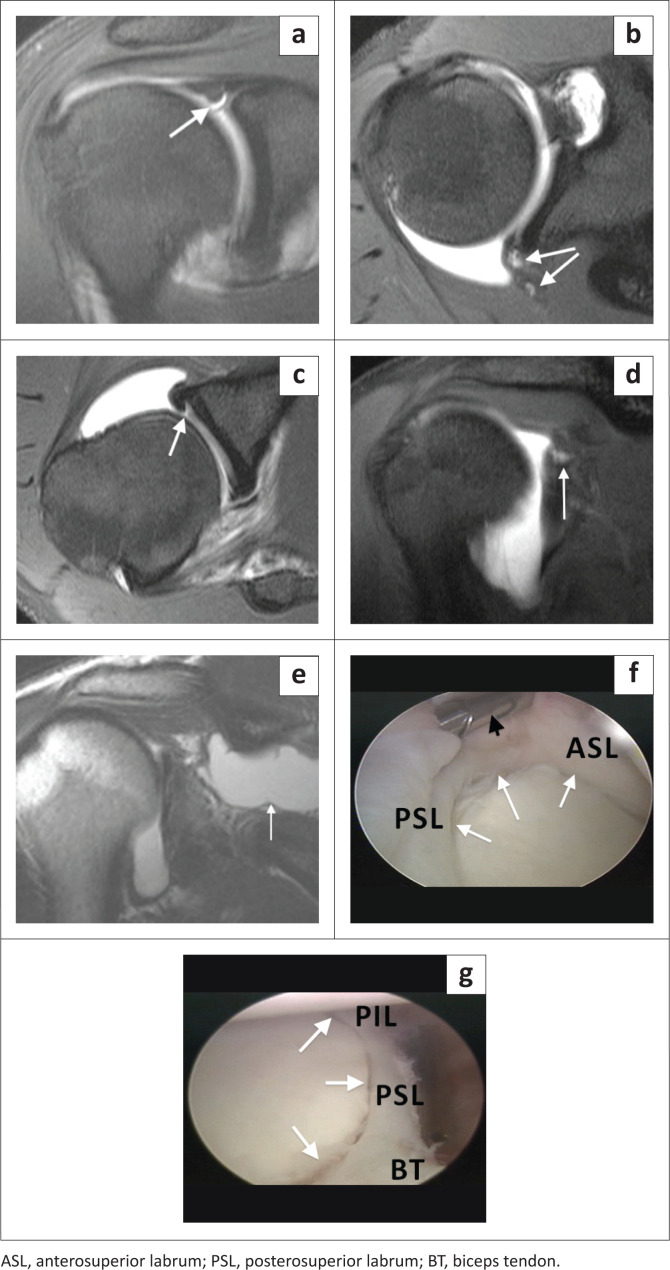
A SLAP type VIII lesion. (a) FS T1-weighted (T1W) MR arthrogram coronal oblique image demonstrating a superior labral tear (white arrow). (b) FS T1W MR arthrogram axial image depicting a tear of the posterior labrum with gadolinium-based contrast filling of the paralabral cysts (white arrows). (c) FS T1W MR arthrogram axial image at the level of the inferior margin of the subscapularis tendon showing the inferior extent of the tear of the posterior labrum (white arrow). (d) FS T1W MR arthrogram coronal oblique image demonstrating a paralabral cyst filling with only a very small amount of gadolinium-based contrast (white arrow). (e) T2-weighted (T2W) coronal oblique image depicting the true size of the paralabral cyst, highlighting the importance of some form of T2W sequence as part of the imaging protocol. (f) Arthroscopic confirmation of a SLAP VIII tear. White arrows indicate the superior labral tear. Black arrow indicates the arthroscopic probe overlying the biceps tendon. (g) Arthroscopic image demonstrating the posterior extension of the tear (white arrows) to involve the posterosuperior (PSL) and posteroinferior labrum (PIL).

The SLAP VIII lesion represents a superior labral tear with extension into the posterior labrum (Figure 12 and Figure 13) but more extensively than that seen in a SLAP type IIB tear (Figure 5a). This tear usually follows a posterior dislocation of the shoulder. The coronal images confirm the superior labral tear and the axial and sagittal images demonstrate the posteroinferior extension of the tear.

**FIGURE 14:**
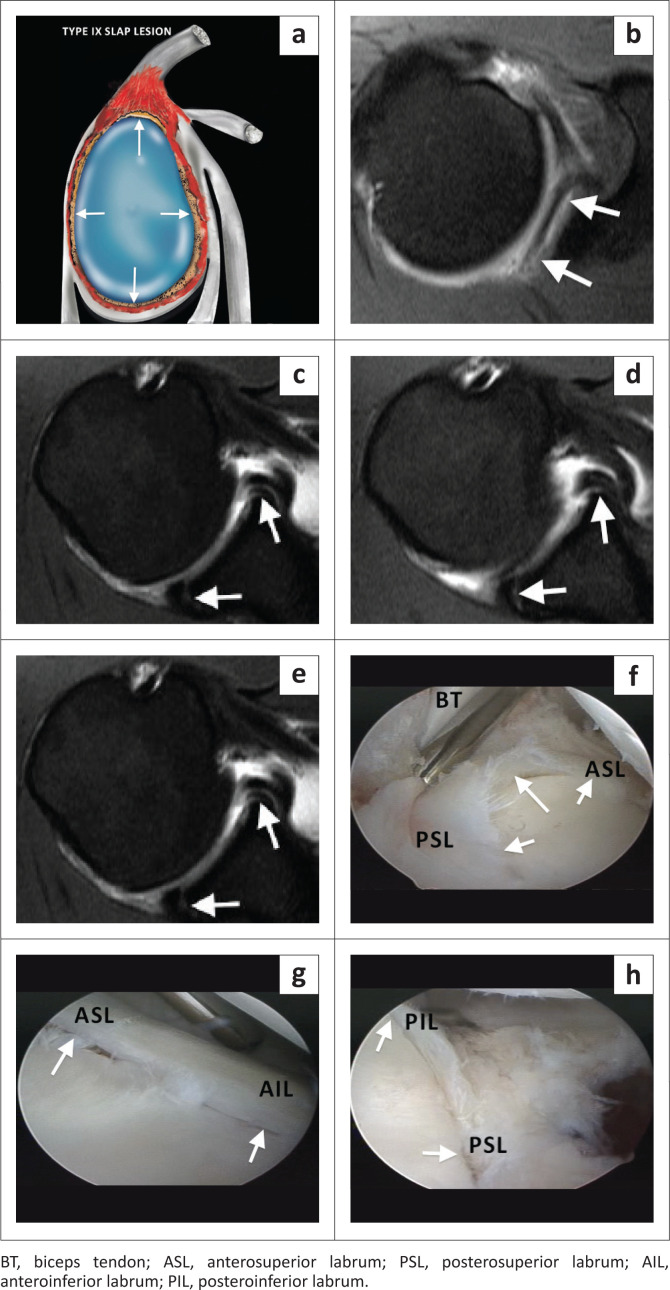
A SLAP type IX lesion. (a) Lateral colour illustration of a SLAP IX lesion. (b–e) Axial images (FS T1-weighted (T1W) MR arthrogram) from superior to inferior through the entire labrum displaying the detachment of both the anterior and posterior labrum (white arrows). (f–h) Arthroscopic confirmation of a circumferential labral tear (white arrows) in this 29-year-old professional rugby player.

Type IX SLAP lesion represents a circumferential labral tear (Figure 14) and is associated with severe acute trauma. The coronal images demonstrate the superior and inferior components of the labral tear, whereas the axial images display the anterior and posterior components of the tear.

**FIGURE 15:**
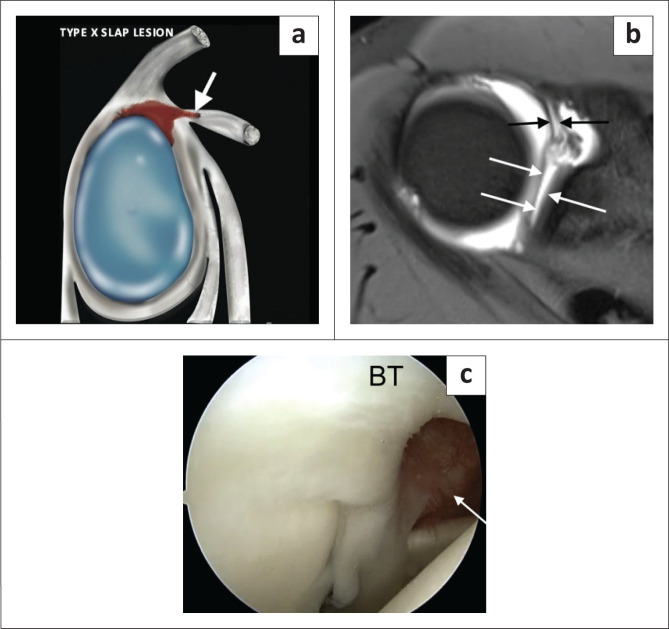
A SLAP type X lesion. (a) Lateral colour illustration of a SLAP X tear. (b) FS T1-weighted (T1W) MR arthrogram axial image depicting full anterior to posterior involvement (white arrows) of the superior labral tear with extension into the superior glenohumeral ligament (SGHL) (black arrows). (c) Arthroscopy confirming a tear of the SGHL (arrow).

Type X SLAP lesion represents a superior labral tear with extension into the rotator interval involving the superior glenohumeral ligament (Figure 15) and usually follows acute severe trauma.

**FIGURE 16:**
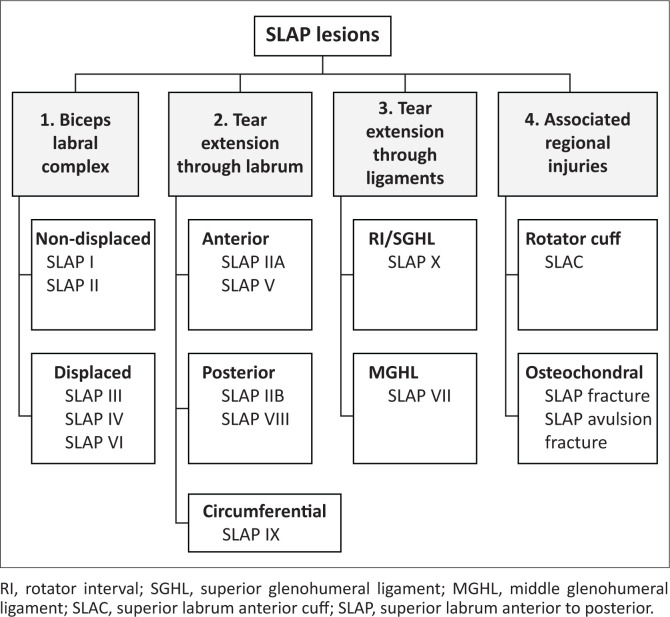
Practical approach to SLAP tear diagnosis on magnetic resonance imaging.

There are several miscellaneous SLAP lesions with the involvement of regional structures. A SLAP fracture represents a SLAP lesion with an associated humeral head chondral fracture, which is typically located superomedially (Figure 1c).^4^ While a rare injury, a SLAP avulsion fracture involves osseous avulsion of the labrum and biceps from the superior glenoid.^4^ A SLAC lesion represents a SLAP II lesion in association with a partial thickness tear of the articular surface of the anterior supraspinatus tendon.^4^ A practical approach to SLAP tear diagnosis on MRI is presented in Figure 16.

## Management

The management of SLAP lesions is controversial, and a detailed review is beyond the scope of this article. The main principle of operative intervention is to restore the stability of the superior labrum and the biceps labral complex. Several factors are taken into consideration including the type of tear, age of the patient, level of activity, stability of the superior labrum and integrity of the biceps anchor as well as associated injuries such as rotator cuff and capsular tears.^21^

**FIGURE 17:**
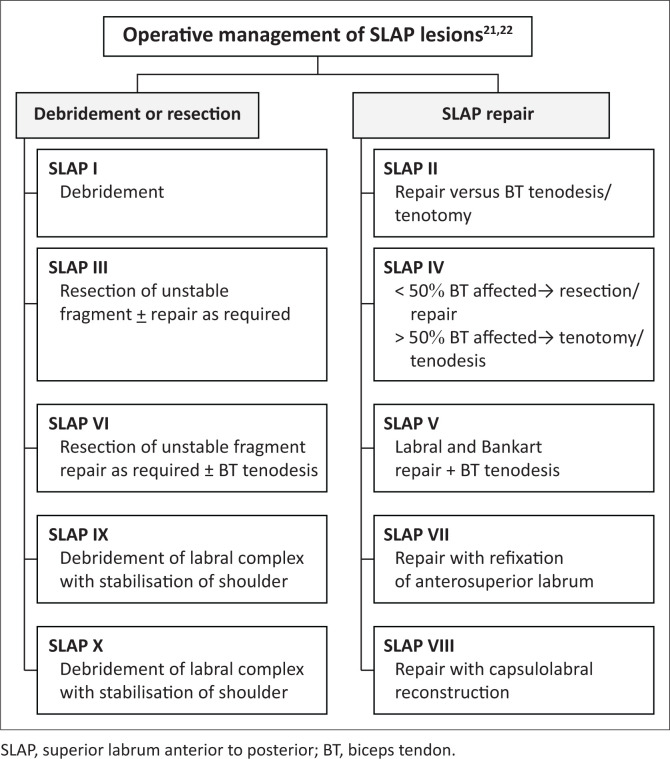
A suggested algorithm for operative management of SLAP lesions.

Conservative management is usually reserved for type 1 lesions. Operative options include labral debridement, repair or resection of the unstable labral fragment. Biceps tendon procedures include debridement, tenotomy and tenodesis. Capsulolabral reconstruction and stabilisation are further options.^21,22^ A suggested management algorithm is highlighted in Figure 17.^21,22^ This underscores the value of MR arthrography as a means of accurate diagnosis and preoperative evaluation in patients with a SLAP lesion.

## Conclusion

Superior labrum anterior to posterior lesions are a common cause of shoulder pain and instability. The clinical diagnosis is difficult and although arthroscopy remains the diagnostic gold standard, MRI, particularly direct MR arthrography, is the optimal imaging modality to evaluate glenoid labral anatomy and pathology and can reliably demonstrate the spectrum of SLAP lesions. A practical imaging approach is recommended that includes evaluation of the biceps labral complex, the morphology of the tear, the extension pattern of the tear to involve other parts of the labrum and glenohumeral ligaments and the associated involvement of the regional structures, which will assist in the management of patients with SLAP lesions.
